# Appropriateness, barriers, and facilitators of multi-month dispensing of tuberculosis drugs in rural eastern Uganda: A qualitative study to inform a non-inferiority randomized trial

**DOI:** 10.1371/journal.pgph.0004539

**Published:** 2025-09-05

**Authors:** Jonathan Izudi, Francis Bajunirwe, Adithya Cattamanchi, Nora West

**Affiliations:** 1 Department of Community Health, Mbarara University of Science and Technology, Mbarara, Uganda; 2 Infectious Diseases Institute, Makerere University, Kampala, Uganda; 3 Directorate of Graduate Training, Research and Innovation, Muni University, Arua, Uganda; 4 Center for Tuberculosis, Institute for Global Health Sciences, University of California San Francisco, San Francisco, California, United States of America; 5 Division of Pulmonary Diseases and Critical Care Medicine, University of California Irvine, Irvine, California, United States of America; 6 Division of Pulmonary and Critical Medicine, San Francisco General Hospital, University of California San Francisco, San Francisco, California, United States of America; Aga Khan University, PAKISTAN

## Abstract

Multi-month dispensing of tuberculosis (TB) drugs is an innovative strategy that may reduce frequent clinic visits and travel costs among people with TB (PWTB) in rural areas. To inform a planned trial, we explored the appropriateness, barriers, and facilitators to multi-month dispensing among PWTB and healthcare providers in rural eastern Uganda. We used qualitative methods situated within the Consolidated Framework for Implementation Research to explore two refill schedules for multi-month dispensing of TB drugs—a four- or five-visit refill schedule. In December 2024, we collected data through interviews with PWTB, their treatment supporters, and healthcare providers at the regional, district, and health facility levels. Data were analyzed using thematic analysis. All participants (n = 39; 22 healthcare providers, 12 PWTB, and five treatment supporters) expressed willingness to adopt multi-month dispensing, with a four-visit schedule as the preferred option. Healthcare providers preferred the five-visit schedule for individuals with complex health conditions: severe illness, clinical instability, or bacteriologically confirmed pulmonary TB. Multi-month dispensing was perceived to benefit healthcare providers by reducing workload, improving patient flow, and enhancing patient management. Perceived benefits to PWTB included reduced clinic visits and travel costs, time savings, improved treatment adherence, reduced wait times and TB-related stigma, and increased satisfaction with care. Facilitators included integration with existing treatment models, person-centeredness, community and family support, reliable drug supply, clear operational guidelines, healthcare provider training and readiness, enhanced monitoring and evaluation, clinic accessibility, readiness to utilize multi-month dispensing, and leadership support. Barriers included undefined eligibility criteria, uncertain effects of multi-month dispensing, differing refill schedules for PWTB and HIV, treatment non-adherence due to forgetfulness and medication sharing, and patient disengagement due to insufficient follow-up. Multi-month dispensing is perceived to benefit PWTB and healthcare providers. Further studies to measure the impact on treatment outcomes should leverage facilitators and address barriers to adoption and effectiveness.

## Introduction

In 2022, the global treatment success rate for drug-susceptible tuberculosis (TB) was 88% [1], and 90.2% in Uganda [2]. In rural eastern Uganda, differences in treatment success rates between and within districts and health facilities are common, with most health facilities having less than a 90% treatment success rate, which is suboptimal [3]. Several factors contribute to suboptimal treatment success rate, including frequent health facility visits and longer travel distances to TB clinics [4,5]. In rural eastern Uganda, travel distances exceeding five kilometers have been associated with poorer treatment outcomes, primarily due to the financial and logistical burden of travel, which increases the risk of missed clinic visits and treatment nonadherence [3,4]. Longer travel distances are associated with missed clinic visits, which in turn reduce treatment success and increase mortality in this setting [6,7]. Missed clinic visits are further exacerbated by the need for frequent travels to health facilities to collect TB medications, as routine care requires eight visits over the six-month treatment period—four in the first two months and four in the remaining four months. Therefore, novel and innovative person-centered strategies are needed to ensure care continuity and improve treatment outcomes among people with TB in this setting.

Among people with human immunodeficiency virus (PWH), multi-month dispensing of anti-retroviral therapy (ART) has been shown to improve viral load suppression, mitigate stigma, reduce the risk of inadvertent HIV status disclosure, and enhance treatment adherence and retention [8–10]. These benefits may be transferable to people with tuberculosis (TB) through multi-month dispensing of TB drugs (MMD-TBD); however, empirical evidence remains limited. We hypothesized that MMD-TBD would not lead to a worse treatment success rate than routine care (bi-weekly dispensing of TB drugs for two months and then monthly for four months). To test our hypothesis, we designed the Multi-Month Refill of Anti-TB Drugs (MORAD) study to evaluate the preliminary effectiveness of MMD-TBD (intervention) compared to routine care in improving treatment success rate among people with drug-susceptible TB aged ≥15 years receiving the standard 6-month anti-TB regimen in rural eastern Uganda.

The MORAD study proposes two options for MMD-TBD (intervention): a four-visit or five-visit refill schedule. The four-visit option would allow monthly TB drug refills for two months, followed by a two-month refill for the next four months. The five-visit option would include bi-weekly refills for the first month, followed by a one-month refill, then a two-month refill for the remaining four months. These refill schedules would be compared with routine care, which involves eight visits: bi-weekly refills for the first two months, followed by monthly refills thereafter. However, there are uncertainties regarding the optimal refill schedule and key barriers and facilitators to MMD-TBD. To inform the randomized trial implementation, we explored the appropriateness, barriers, and facilitators to MMD-TBD among people with drug-susceptible TB in rural eastern Uganda. Specifically, we assessed the perceived appropriateness, barriers, and facilitators of MMD-TBD among people with drug-susceptible TB, healthcare providers, and TB treatment supporters in rural eastern Uganda.

The MORAD study protocol was previously published [11] and registered with the Pan African Clinical Trials Registry (PACTR202403586718783). In this study, we utilize the abbreviation ‘MMD-TBD’ (Multi-Month Dispensing of TB drugs) to refer to providing TB drugs for multiple months. The abbreviation ‘MMD-TBD’ replaces ‘MULTI-DAT’, which was used in our previous protocol and conveys the same concept. Here, ‘MMD-TBD’ was used instead of ‘MULTI-DAT’ to maintain clarity and consistency with prior studies [12,13] that employed ‘MMD’ to mean multi-month dispensing.

## Materials and materials

### Ethical issues

The MORAD study received ethical approval from the Infectious Diseases Institute Research Ethics Committee (reference number: IDI-REC-2023–33) and the Uganda National Council for Science and Technology (reference number: HS3953ES). Administrative clearances were granted by the National TB and Leprosy Control Program at the Uganda Ministry of Health and all District Health Offices. Participants gave written informed consent after being fully informed about the study’s objectives, benefits, risks, privacy measures, confidentiality, and protections, including their right to withdraw at any time.

### Study setting, design, and population

This study was conducted in four districts in rural eastern Uganda, namely Soroti, Kumi, Ngora, and Serere. Serere and Ngora districts reported optimal treatment success rates exceeding 90%, despite observed inter- and intra-facility variations. In contrast, treatment success rates in Soroti and Kumi districts remained below 90%, with similar variability across and within health facilities. These districts have varying levels of health facilities, including health center (HC) IIs, IIIs, IVs, and hospitals. Soroti and Kumi have district hospitals, while Ngora and Serere do not have district hospitals.

Across the districts, all hospitals and HC IVs were included in the study because they serve a larger number of people with TB and offer comprehensive TB services. Specifically, we included three high-level facilities from Soroti district (one hospital and two HC IVs), three health facilities from Kumi district (two hospitals and one HC IV), two from Ngora district (one private hospital and one HC IV), and two from Serere district (two HC IVs). Each health facility has a TB clinic. This qualitative study was conducted in 10 TB clinics across the four districts.

In this region, the majority of people with TB travel five or more kilometers to access TB care [3,4]. The 10 health facilities treat 50 or more people with TB annually. Each health facility has a TB clinic managed by a TB focal person—an experienced medical, clinical, or nursing officer. The clinics follow the National TB Control Program guidelines for standardized TB care. The participants included people with TB, their treatment supporters, and healthcare providers (HCPs) at the health facility, district, regional, and national levels. All HCPs had ≥ 3 years of TB work experience. Eligible people with TB were those who had received TB treatment for ≥4 months, along with their respective treatment supporters. These individuals were considered as they have a comprehensive understanding of TB treatment requirements based on their prior experience with TB care, and they provided valuable insights into the proposed MMD-TBD intervention. TB focal persons included people who had provided TB care for ≥1 year.

The HCPs were purposively selected as key informants, while people with TB were consecutively sampled. Treatment supporters were recruited using a snowball sampling method. Here, people with TB who participated in the study invited their treatment supporters to contribute insights upon request.

### Data collection and study variables

Between December 2, 2024, and December 22, 2024, qualitative data were collected through key informant interviews (KIIs) with HCPs at their workplace and in-depth interviews (IDIs) with people with TB and their treatment supporters at TB clinics. S1 File shows the interview guides. The Consolidated Framework for Implementation Research (CFIR) [14,15] guided the data collection. Details of the CFIR domains are described previously [11]. In brief, we collected data on the intervention characteristics, outer setting, inner setting, characteristics of individuals, and implementation process. Additionally, we explored the acceptability and appropriateness of the implementation strategy, including the rationale. KIIs were conducted in the English language, while IDIs were held in the local language *(“Ateso”).* All research assistants had ≥ 5 years of qualitative research experience and held, at a minimum, a diploma in health sciences.

Field notes were taken during the data collection to capture additional details beyond the verbal responses of the participants. For example, the field notes captured non-verbal cues and researcher reflections, and were used to complement and enrich the interview data. Each interview lasted 15–35 minutes, on average.

### Quality control measures

We pre-tested and validated the data collection tools outside the study area to ensure reliability. Research assistants received comprehensive training on ethical research practices, the study protocol, participant recruitment, and data collection procedures. Audio recordings were transcribed within five days of data collection to maintain accuracy, and a Research Team Leader oversaw the research team, reviewed transcripts, and ensured alignment with study objectives. The Research Team Leader was independently recruited to supervise the research assistants and did not participate in data collection.

### Sample size and data analysis

Sample size depended on the saturation principle, a point at which no new information emerged even when additional data were collected [16]. However, given the interviews were focused on a specific topic of interest among stakeholders, the *a priori* sample size estimated to reach the saturation point was 66 participants—three participants at the Ministry of Health (MoH) or national level, one participant at the regional level, 12 participants at the district level, and 50 participants (10 TB focal persons, 20 persons with TB, and 20 treatment supporters) at the health facility level. The study team reviewed the audio recordings, field notes, and transcripts during the data collection process to determine when saturation was met. At the regional level, only one participant (the Regional TB and Leprosy Supervisor) was available, and was therefore purposively selected and included in the study.

We conducted a thematic analysis to identify the appropriateness, barriers, and facilitators to implementing MMD-TBD grounded within the CFIR domains. Audio recordings were transcribed verbatim, and the emergent transcripts were verified by re-reading and replaying the audio recordings, while correcting any discrepancies. Two analysts (JI and NW) familiarized themselves with the data by reading about 10 transcripts multiple times. An initial codebook was developed based on the domains, and consensus was established through discussion. The remaining transcripts were coded using inductive and deductive methods, guided by the initial codebook. Inductive coding allowed us to generate codes directly from the data without relying on pre-existing CFIR categories, thus capturing context-specific insights. In contrast, deductive coding involved applying codes derived from the established CFIR domains before data analysis, ensuring alignment with our conceptual framework. The use of a hybrid approach provided a balance between theoretical structure and openness to new, emergent themes.

JI is a public health specialist with 10 years of experience in mixed-methods research. NW is a socio-behavioral scientist with nearly 12 years of qualitative research expertise. Both JI and NW hold doctorates in public health and conducted the data analysis independently. Sub-themes were iteratively developed based on the CFIR domains, with non-representative sub-themes discarded. Two senior analysts (FB and AC) conducted the final review of the sub-themes within the CFIR domains to ensure they accurately reflected the data and study objectives. Results were reported using themes supported by illustrative participant quotes within the CFIR domains (S2 File). Ethical rigor was maintained through member-checking, reflexivity to reduce subjective bias, an audit trail, and data triangulation. To ensure transparency and methodological rigor, we adhered to the Consolidated Reporting of Qualitative Studies (COREQ) guideline (S3 File) [17].

### Inclusivity in global research

Information regarding ethical, cultural, and scientific considerations specific to inclusivity in global research is included in the Supporting Information (S1 Checklist).

### Reflexivity, positionality, and rigor

The research assistants were from the study setting, held diplomas in health sciences, and had at least three years of qualitative research experience. They were trained on interview techniques, ethics, and reflexivity to minimize bias and power imbalances during the data collection. Additionally, the team had backgrounds in public health and TB/HIV care and regularly reflected on their positionality and potential influence on the research process. We used field notes and analytic memos to document emerging insights and assumptions, including reflexive team discussions during coding to challenge interpretations and reduce bias. To ensure rigor, we triangulated data sources and held peer debriefings (credibility), provided detailed contextual descriptions (transferability), and maintained an audit trail of coding and analytic decisions (dependability). In addition, we applied reflexive journaling focused on preconceptions about the data and the role of the interviewers and analysts in interpreting and shaping findings, to enhance transparency and reduce bias (confirmability).

## Results

### Characteristics of participants

The study involved 39 participants across four districts, as shown in Table 1. Most (59.0%) of the participants were from a health facility level, and 64.1% were male. Participants’ ages ranged from 24 to 62 years, with most (82.1%) falling within the 35–59 age category. Of the participants, 22 (56.4%) were HCPs, and 17 (77.3%) had at least 10 years of work experience. Although we initially planned to interview three participants (senior technical officers) at the national level, these interviews were not conducted. Data from district-level senior TB technical experts (District Health Officers, District TB and Leprosy Supervisors, and District Laboratory Supervisors), together with information from Regional TB and Leprosy Supervisors, were sufficient to achieve thematic saturation.

**Table 1:** Summary of participant characteristics.

Variables	Level	Overall (n = 39)
District	Kumi	14 (35.9)
	Ngora	7 (17.9)
	Serere	7 (17.9)
	Soroti	11 (28.2)
Administrative unit	District	15 (38.5)
	Health facility	23 (59.0)
	Regional	1 (2.6)
Sex	Female	14 (35.9)
	Male	25 (64.1)
Age categories (years)	24–34	6 (15.4)
	35–59	32 (82.1)
	60 and over	1 (2.6)
	mean (standard deviation, SD)	42.0 (8.6)
Type of participants	HCPs	22 (56.4)
	People with TB	12 (30.8)
	Treatment supporter	5 (12.8)
Highest level of education	None	3 (7.7)
	Primary	6 (15.4)
	Secondary	6 (15.4)
	Diploma	12 (30.8)
	Bachelor’s degree	9 (23.1)
	Master’s degree	3 (7.7)
Work experience (years, n = 22)	Less than 10	5 (22.7)
	10 and over	17 (77.3)
	mean (standard deviation, SD)	16.6 (8.8)
Data collection approach	In-depth Interviews	17 (43.6)
	Key informant interviews	22 (56.4)

### Timing and frequency of MMD-TBD

MMD-TBD was perceived as acceptable by all participants. HCPs unanimously supported a two-month TB drug refill schedule during the continuation phase, requiring only two visits. They also agreed to modify TB refills during the intensive phase to allow two or three visits. They recommended the 4-visit schedule for MMD-TBD for stable individuals—those with non-severe forms of TB and people with clinically diagnosed pulmonary TB.

*“When that patient has two visits in the first month [bi-weekly TB drug refills], and if that person is doing well, then that person can be given monthly refills to finish the initial phase. And then in the continuation phase, can now be given, every 2 months.” (District Health Officer, D1, Male).*

The 5-visit schedule for MMD-TBD was recommended for people with TB considered physically weak or unstable, as well as those with bacteriologically confirmed pulmonary TB. They stated that the first two weeks of the intensive phase of TB treatment would allow HCPs to assess the stability of people with TB, including monitoring for side effects and determining their readiness for MMD-TBD.

*“I think from the initial start, we give them drugs for one month. That is only if the patient is stable. But if the patient is not stable, that would require the patient to come back in the first two weeks again. For the continuation phase, we can give the two monthly refills.” (TB focal person, D2, Male)*

Furthermore, participants favored the five-visit schedule, citing the need for close monitoring of TB drug-related side effects and treatment adherence. MMD-TBD was perceived as appropriate once a person with TB showed no side effects and demonstrated good adherence. An illustrative quote supporting this argument was:

*“I think the proposal is good to have five [visits] refills in the course of [TB] treatment. Like the intensive phase, the first month of picking drugs every two weeks should remain because of the side effects of the drug on the patient and treatment adherence. Then the last four months, we can give the medications twice.” (TB focal person, D3, Female).*

Another reason for the five-visit schedule was the need for close clinical monitoring of people with TB until their condition improved. Participants emphasized that initiating MMD-TBD should only occur once clinical improvement was achieved and consistent treatment adherence was established:

*“I agree with the schedule stating that in the intensive phase, someone [people with TB] should come every two weeks for the first month. …….we need to know how are they responding to medicines. Whether they are getting any complications and whether they are taking medicines”. (TB focal person, D4, Male).*

### Perceived benefits of MMD-TBD for people with TB and HCPs

We summarized the benefits of MMD-TBD for people with TB and HCPs in Table 2. The perceived benefits included: 1) reducing the frequency of clinic visits, resulting in time and cost savings for people with TB and a reduced workload for HCPs; 2) improving the TB treatment experience and outcomes by making drug refills more convenient, reducing TB-related stigma, and enhancing treatment adherence; 3) reducing wait times and clinic congestion due to less frequent visits; and 4) enhancing patient satisfaction with TB care by making treatment less burdensome.

**Table 2:** Summary of perceived benefits of MMD-TBD for people with TB and HCPs.

People with TB	Healthcare providers (HCPs)
**1. Reduced frequency of clinic visits saves time, costs, and reduces workload**	
People with TB perceived that MMD-TBD would reduce the frequency of clinic visits, saving time and money on transport.	MMD-TB would result in fewer visits, hence lowering the workload on HCPs.
*“Every time a patient visits a health center, it is money (cost), and that money may not be there. You may find that the patients keep dodging (missing) clinic appointments because they cannot come.” (District Health Officer, D1, Male).*	*“I think if we come here a few times, even the health workers will not have a lot of work [reduced workload] because instead of every two weeks, patients will be coming once a month. So, once we do that, I’m sure even the health workers will not get bored of seeing us, the patients.” (Treatment supporter, D3, Male).*
**2. Improved TB treatment experience and outcomes**	
MMD-TBD was perceived as improving convenience and reducing exposure to stigma	HCPs believed that MMD-TBD would improve treatment adherence and treatment outcomes by reducing defaulting and allowing more time for high-need patients.
*“When I am given drugs for many months [MMD of TB drugs], my business will not collapse because I will have time for it, since the frequency of coming to the facility [health facility] will reduce if I am given 2 months.” (PWTB, D1, Female).*	*“It [MMD of TB drugs] will improve adherence and has been long overdue. So, this [routine care] has been causing defaulting in treatment, even adherence, because sometimes some of these patients stay alone or with no caregivers. Coming to the health facility frequently will lead to someone even abandoning their treatment.” (TB focal person, D3, Female).*
*This [MMD of TB drugs] will help me because some of my friends have started to tease me. They have made me fear coming to collect the drugs. But, when it is reduced, it will save me the time and that fear”. (PWTB, D2, Female).*	*“We are understaffed, and having these patients visit a facility fewer times would give health workers much of time to concentrate on the other areas of patient care.” (Laboratory focal person, D3, Male).*
**3. Reduced wait times and clinic congestion**	
People with TB reported long queues and delays during frequent clinic visits, which MMD-TBD would help reduce.	
*“I come here [health facility] and find the line is long [long queue] and I have to wait the whole day [longer waiting time]. And then I have to do that again in two weeks. So, it would be better if we did [travel to the health facility] once a month.” (Treatment supporter, D2, Male).*	
**4. Enhanced satisfaction with TB care**	
People with TB expressed that fewer visits would increase their satisfaction with care by making it less burdensome.	HCPs felt MMD-TBD would make TB care more acceptable and increase the willingness of people with TB to stay on treatment.
	*“The truth is that patients will be very happy to know that this treatment [TB treatment] no longer requires frequent visits if we start giving the TB drugs every two months”. (TB focal person, D2, Male)*

### Facilitators and barriers to MMD-TBD based on the CFIR domains

The emergent facilitators and barriers to MMD-TBD, categorized according to the CFIR domains, are summarized in Table 3. Our analysis identified multiple facilitators across all five CFIR domains; however, no barriers were identified within the inner and outer settings domains.

**Table 3:** Summary of facilitators and barriers to MMD-TBD based on the CFIR domains.

CFIR domains	Facilitators	Barriers
Intervention characteristics	• Integration with existing treatment models • Person-centeredness of MMD • Practical supporting evidence	• Undefined eligibility criteria for MMD • Uncertain effects of MMD • Differing refill schedules for people with TB/HIV co-infection
Outer setting	• Presence of community health workers for decentralized support • Family-level treatment support and engagement	
Inner setting	• Steady availability of drugs • Operational guidelines (MMD protocols and procedures) • Medication instructions • Enhanced monitoring and evaluation (e.g., revised data collection tools, regular reviews) • Clinic accessibility for managing medication side effects and complaints	
Characteristics of individuals	• HCP training and mentorships • HCP readiness to implement MMD • Patient motivation and readiness to utilize MMD	Non-adherence due to forgetfulness, medication sharing, and health neglect
Process of implementation	• Patient engagement and support (e.g., active follow-up, patient reminders, health education, and counseling) • Leadership support for MMD implementation	Patient disengagement driven by insufficient follow-ups

### Facilitators of MMD-TBD based on the CFIR domains

#### Intervention characteristics.

***Integration with existing treatment models*:** HCPs reported that MMD-TBD aligns well with existing treatment models, such as the MMD of ART and anti-hypertensives. Specifically, they noted that the MMD approach is already used to deliver ART to PWH and other patients, making the introduction of MMD-TBD consistent with existing MMD guidelines:

*“If you look at the schedule, at least you see a patient four to five times in six months, which I think is good. And, also, I think it aligns well with the multi-month schedule for HIV, which we are doing now.” (TB focal person, D1, Male).*

***Person-centeredness of MMD*:** Participants, particularly HCPs, stated that MMD-TBD embodies a person-centered care approach by empowering individuals with TB to manage their treatment. They emphasized that its person-centeredness would facilitate/ or enable the adoption and utilization:

“*To the clients [people with TB], this approach [MMD of TB drugs] is patient-centered care, so the issue of transport costs is reduced. And, we give clients the responsibility to take care of themselves away from us [the HCPs] a little.” (TB focal person, D4, Male).*

***Practical supporting evidence*:** It emerged that practical evidence supporting MMD was available, with some HCPs indicating its informal use in current practice. Based on the informal use of MMD-TBD, HCPs stated that the approach was effective during and after the COVID-19 pandemic.

*“I have tried [MMD of TB drugs] although it was not in the treatment guideline. But we have been trying, and you find the books [prescriptions] would get rejected. You would find the dispenser trying to reduce the period. (TB focal person, D2, Female).*

#### Outer setting.

***Presence of CHWs for decentralized support*:** The availability of community health workers (CHWs), including Village Health Team (VHT) members, was recognized as a crucial facilitator of decentralized, family-level treatment support. Participants emphasized that CHWs could enhance adherence to directly observed therapy (DOT).

“*Another option for follow-up can be the use of the VHTs [Village Health Teams]. So, the nearby VHT can follow up on this patient and then compile the reports and send them to the health center every month.” (District Health Officer, D4, Male).*

***Family-level treatment support and engagement*:** A strong family involvement in treatment adherence was highlighted as an essential facilitator for the successful uptake and implementation of MMD-TBD. Participants underscored the importance of engaging family members in supporting adherence to optimize treatment outcomes.

*“As a mother who is giving the drugs to my child, I will ensure the child takes the drugs on time. If I am not at home, I will call someone at home to ensure the child is given the drugs on time because it will not be good for his health.” (Treatment supporter, D4, Female).*

### Inner setting

***Steady availability of drugs*:** The consistent availability of TB drugs was identified by nearly all HCPs and some people with TB as a key facilitator of MMD-TBD, ensuring uninterrupted medication refills and supporting treatment adherence.

*“The most important thing is having the drugs available for it [MMD of TB drugs] to be successful. We must have enough stock available for us to be able to successfully implement it.” (TB focal person, D3, Male).*

***Operational guidelines (MMD protocols and procedures)*:** The development of operational guidelines, including standardized protocols and procedures for MMD implementation, was identified as a key facilitator. At the health facility level, participants emphasized the need for a standard operating procedure (SOP), while at the national level, they stressed that the need for national guidelines to support MMD-TBD:

*“The way I know, it will be like a policy. The Ministry of Health [MoH] has to produce a standard operating procedure (SOP) whereby health workers are supposed to follow” (TB focal person, D3, Male).*

***Medication instructions*:** Clear medication instructions stating the frequency of drug pick-ups were considered an essential facilitator of MMD-TBD by some HCPs and treatment supporters, but not by people with TB.

*“Maybe they [HCPs] can give us some write-up [medication instructions] so that we can also be reading on what you’re supposed to be doing.” (Treatment supporter, D2, Male).*

***Enhanced monitoring and evaluation (e.g., revised data collection tools, regular reviews)*:** Enhanced monitoring and evaluation through updated data collection tools, such as a revised TB register to differentiate people with TB who are on MMD from those on routine care, emerged as a facilitator for MMD-TBD.

*“I think for those selected health facilities [for MMD-TBD], the tools [data collection tools] equally need to be provided. So, let those columns in the TB Unit register for client visits be changed to fit MMD.” (TB focal person, D2, Female)*

Additionally, HCPs emphasized a need for regular data reviews as a necessary facilitator for generating evidence on the performance of MMD-TBD. They noted that if MMD-TBD proves effective, such evidence would encourage more people with TB to adopt it.

*“There should be monthly monitoring of data to see who has not come and who has not come. At the monthly data review, these data should be shared or presented to all staff to know that there are patients on treatment and that new changes [MMD of TB drugs] have happened.” (TB focal person, D2, Male)*

***Clinic accessibility for managing medication side effects and complaints*:** HCPs and people with TB stated that there may be medication-related side effects and other health concerns among individuals initiated on MMD-TBD. They emphasized the need for continuous access to TB clinics to ensure timely management and support.

*“I think the health facility should constantly be open to us [accessible health facility] so that if we [people with TB] take these drugs and develop a problem [side effects], I should be able to come back to the health facility at any time for the health workers to see me.” (PWTB, D2, Female).*

#### Individual characteristics.

***HCP training and mentorships*:** The training of HCPs on MMD-TBD was identified as a key facilitator. Participants highlighted that the training would ensure effective MMD implementation, including clarity about its relevance and benefits.

*“Before this program takes place [wide implementation of MMD of TB drugs], we need to organize either at the district level or at the health facility level, serious mentorships or training of the focal persons, plus even the clinical team on TB management.” (TB focal person, D4, Male).*

***HCP readiness to implement MMD*:** The readiness of HCPs to implement MMD emerged as an important facilitator. Participants specifically highlighted the presence of TB focal persons (HCPs overseeing TB service delivery) and their readiness as being crucial for the successful implementation of MMD-TBD.

*“We have no problem with it [MMD of TB drugs] because it is very good on the patient side and very good on the health worker side. At the health facility, the TB focal person is already there to track these patients. (District Health Officer, D4, Male).*

***Patient motivation and readiness to utilize MMD*:** The readiness of people with TB to adopt MMD-TBD, as demonstrated by their high motivation, was identified as a key facilitator. HCPs reported that people with TB often expressed a preference for MMD-TBD, but they could not help as the approach was not formalized.

*“Even as patients [people with TB], we have been thinking about it [MMD of TB drugs], but we have nowhere to take the idea.” (PWTB, D2, Female)*

Similarly, treatment supporters were receptive to MMD-TBD and noted that the implementation was long overdue.

*“I will be happy with the initiative [MMD of TB drugs] and even propose that they increase it to 3 months so that in a short time I will finish collecting the drugs [TB drugs].” (Treatment supporter, D2, Male)*

#### Implementation process.

***Patient engagement and support*:** All HCPs indicated that the active engagement of people with TB, including supporting them throughout the implementation of MMD-TBD would be crucial facilitators. Key modalities for achieving patient engagement and support included active follow-up by HCPs, sending reminders, and providing adequate health education and counseling. Specifically, phone calls and health education/counseling were identified as critical strategies to enhance treatment adherence.

*“We need to strengthen patient education at the facility level. Let the patient understand that at the end of 1 month, he/she is supposed to come for a refill. We need proper counseling of patients [people with TB] so that they can follow up on the treatment very well.” (TB focal person D3, Female)*

Additionally, documenting all MMD-TBD schedules to ease tracking was highlighted as a key facilitator for successful implementation.

*“We [HCPs] need to do proper documentation and even put up a schedule since these patients are few. We need to draw up a schedule of visiting clients [people with TB] at regular times based on the refills.” (Laboratory focal persons, D3, Female)*

***Leadership support for MMD implementation*:** Participants emphasized the need to engage leaders at all levels—national, regional, district, and health facility—to ensure the effective implementation of MMD-TBD. At the district level, the key stakeholders included TB focal persons (HCPs overseeing TB service delivery) and the district TB team. At the regional and national levels, engagement should include Regional TB Focal Persons (HCPs overseeing TB service delivery at the regional level) and the National TB Control Program.

*“We need to bring the leaders on board. And, when I look at the letters [support letters from NTLP and the districts] and the people being interviewed, they are the people at the heart of TB management. So, the research is already giving us a direction. I feel that the same approach should be used up to the lower level so that the health facility heads [In-charges] are brought on board.” (TB supervisor, Male).*

### Barriers to MMD-TBD based on the CFIR domains

Several barriers to MMD-TBD were identified. No barriers were found within the inner and outer settings of the CFIR domains.

#### Intervention characteristics.

***Undefined eligibility criteria for MMD*:** Key barriers related to the intervention characteristics included undefined eligibility criteria for MMD-TBD. HCPs argued that MMD may not be appropriate for all people with TB.

“*All in all, giving a multi-month refill is not a bad idea, but then to which category of people are we giving a multi-month refill? I would wish that we first put a specific category of patients on monthly refills and not generalize to all.” (TB supervisor, D1, Male).*

One criterion highlighted by HCPs was the immediate initiation of MMD-TBD for individuals with clinically diagnosed pulmonary TB and a one-month delayed initiation for those with bacteriologically confirmed pulmonary TB. They argued that delayed initiation of MMD-TBD would allow people with bacteriologically confirmed pulmonary TB to receive adequate treatment support and health information to ensure optimal treatment adherence.

*“We should start multi-month treatment for PCDs [persons with clinically diagnosed pulmonary TB] and keep the PBCs [persons with bacteriologically confirmed pulmonary TB] at the health facility because they are very infectious.” (TB supervisor, D3, Male).*

***Uncertain effect of MMD*:** HCPs expressed concerns about the unknown effect of MMD-TBD in improving treatment adherence and outcomes compared to routine care and called for a randomized trial before its implementation.

*“You need to evaluate it. Is it doable? Is it helping? You need to make a comparison between the new approach [MMD of TB drugs] and the present one. You need to follow it up to see if it is working [asses effectiveness]. “(Laboratory focal person, D2, Female).*

***Differing refill schedules for people with TB/HIV*:** For people with TB/HIV, differences in refill schedules for ART and TB drugs emerged as an additional barrier; however, this was considered manageable, as both MMD-TBD and ART refills can be aligned.

*“What is again important is to harmonize TB and HIV refills so that the pill burden and the appointment date match. Because, you know, most TB drugs are packed for three months and one month, and most of the health facilities I have visited have ARVs packed for three months.” (TB supervisor, D2, Male)*

#### Characteristics of individuals.

***Non-adherence*:** Treatment nonadherence emerged as a significant barrier within the CFIR construct of individual characteristics. The main reasons for non-adherence included the following:

a. **High pill burden**

Major factors contributing to nonadherence included perceived treatment burden, as people with TB may not adhere to their medications due to a high pill burden.

*“The patient [person with TB] may mean to relax when they get too many drugs at ago. The patient now starts negotiating within him or herself.” (HCP #13, D2, F).*

b. **Alcohol consumption**

Concerns were raised by some HCPs about alcohol consumption among people with TB enrolled on MMD-TBD.

Locally brewed alcohol is widely sold in rural eastern Uganda as an alternative source of livelihood, and many people with TB consume alcohol. This could hinder treatment adherence, as the MMD-TBD approach reduces the need for frequent visits to health facilities.

“*One of the worst things that might affect [MMD of TB drugs] will be alcohol drinking because somebody will even forget to take drugs.” (Laboratory focal person, D3, Male).*

However, some HCPs and treatment supporters suggested that health education or counseling on the dangers of alcohol consumption could help reduce alcohol consumption. An illustrative quote supporting this claim is below:

*“With such people [alcoholics], we just need information [health education or counseling]. We give them more information, encourage them, and let them know that we are proposing to reduce the number of times to go to the hospital to collect drugs.” (Treatment supporter, D4, Male).*

c. **Forgetfulness**

A few HCPs mentioned that the MMD-TBD could lead to treatment non-adherence, as people with TB might forget to take their pills. They indicated that, since the pills would be available for a longer period, it may not be obvious to people with TB that they need a refill.

“*There’s a tendency to forget to take the drugs once you have all of them [TB drugs]. You will forget to swallow them.” (TB supervisor, D4, Female).*

d. **Medication sharing**

Participants noted that medication sharing among people with TB exists, although it is not very common. Particularly, they said that the tendency to share medications arises when patients receive refills that last for longer periods.

*“When some patients are given more drugs, they start to share them with other people or use them for other things.” (TB focal person, D1, Female).*

e. **Health neglect**

Some people with TB suggested that a few of their colleagues have neglected their health by not taking medications, even when refilled for longer periods. They proposed that ongoing counseling and encouragement could help reduce this neglect.

*“Someone may not take the drugs once given. This is because some people are careless and do not love themselves. But when we have someone at home to keep advising and encouraging the person, the person will take it.” (PWTB, D1, Female).*

#### Implementation process.

***Patient disengagement driven by insufficient follow-ups*:** Within the implementation process, patient disengagement due to insufficient follow-up was identified as a barrier. Participants noted that inadequate follow-up for people on MMD-TBD could result in more frequent loss-to-follow-up and negatively affect treatment outcomes.

*“This [MMD] needs some careful implementation and follow-up so that it does not lead us to high loss to follow-up and [treatment] failure rates. Without the proper follow-up of these patients, they may come back when they are worse than the way we started [them on treatment].” (District Health Officer, D1, Male).*

## Discussion

This qualitative study was designed to inform a non-inferiority randomized trial on refill scheduling and assess the appropriateness, barriers, and facilitators of implementing MMD-TBD in rural eastern Uganda. The findings revealed a consensus among participants on the need to adopt MMD-TBD. The four-visit schedule was the most preferred option, while the five-visit schedule was recommended by HCPs for people with complex health conditions, including those who are severely ill, clinically unstable, or diagnosed with bacteriologically confirmed pulmonary TB. These data support further evaluation of MMD-TBD as a person-centered strategy to improve TB treatment outcomes. Longer TB drug refills are increasingly being considered a best practice to ensure uninterrupted treatment, even for patients requiring more frequent clinic visits due to clinical disease severity. For instance, a 2-month TB drug refill during the intensive treatment phase has been proposed to support treatment completion [18].

Our study showed that MMD-TBD is perceived to offer several benefits for both HCPs and people with TB. For healthcare providers, MMD-TBD reduces workload, saves time, and enhances patient management capacity. For people with TB, benefits include reduced travel costs, increased convenience, minimized stigma, and improved treatment adherence and satisfaction. These findings suggest that MMD-TBD addresses core challenges in rural TB care delivery, where geographic barriers and resource constraints significantly impact treatment access and continuity.

To the best of our knowledge, no published study has focused on MMD-TBD. However, our findings are consistent with several studies of MMD of ART conducted among PWH. These studies have shown that MMD of ART decongests health facilities, reduces HCP workload, alleviates HCP work burnout [13], and improves health service delivery and ART adherence [19]. MMD of ART for up to 6 months has also been shown to significantly reduce HIV clinic volumes and enhance access to treatment by improving care continuity and reducing patient travel burden [20]. Similarly, MMD-TBD may help address health system challenges and alleviate the burden on affected individuals and families [21].

The alignment of MMD-TBD with existing treatment models and person-centered care approaches emerged as primary facilitators. These facilitators resonate with findings from studies on anti-hypertensives, which also highlight patient-centered care as beneficial for stable individuals [22]. Findings around HCP training, data review, HCP readiness to start MMD-TBD, and patient engagement and support systems, such as active follow-up, reminders, health education, and counseling, are consistent with a previous study on a six-month MMD of ART implementation [23]. The strong social support systems characteristic of rural eastern Uganda, including community health workers (CHWs) and family treatment supporters, represent significant assets for MMD-TBD success, which aligns with previous studies in Uganda highlighting a strong social support system, including treatment supporters, for achieving optimal treatment success [3,24]. HCP training and their readiness to implement MMD-TBD, along with the willingness of people with TB to engage in MMD-TBD, were identified as key facilitators. These findings are consistent with a study among PWH, which reported an increasing demand for MMD as a significant facilitator [25].

Leadership support and clear operational guidelines with enhanced monitoring systems were identified as crucial factors in facilitating MMD-TBD implementation. This is consistent with the pivotal role of leadership and guidelines in successfully expanding access to TB drugs through MMD as evidenced by PEPFAR’s partnership with Ministries of Health during the COVID-19 pandemic response [26]. While previous studies do not directly support these findings, effective data collection and analysis are crucial for evaluation. Tools like TB unit registers enable accurate documentation, and regular reviews aid progress tracking. These have improved the uptake of MMD of ART in Tanzania [25]. The convergence of perspectives between HCPs and people with TB regarding benefits and implementation considerations suggests broad stakeholder support, creating a favorable environment for MMD-TBD introduction.

Several barriers emerged that reflect the unique complexities of TB care in rural Uganda. Unclear eligibility criteria and uncertain long-term effects, create hesitation among HCPs, while mismatched refill schedules for people with TB/HIV present operational challenges. Adherence-related challenges such as pill burden, alcohol use, forgetfulness, medication sharing, and health neglect were identified, alongside logistical issues such as drug stockouts and missed appointments due to misaligned dispensing schedules. These barriers align with findings from previous studies involving PWH, which noted challenges related to medication storage and infrequent health monitoring [13]. While improved drug availability is essential, risks such as stockouts and logistical issues like missed appointments due to misaligned dispensing and testing schedules are documented challenges [19,23].

Notably, HCPs identified medication sharing and storage as barriers; however, people with TB and their treatment supporters reported no such concerns, highlighting a discrepancy between perspectives [21]. Barriers such as patient disengagement, primarily from inadequate follow-up and treatment non-adherence, significantly impact treatment outcomes among people with TB in low-resource settings [27] but can be mitigated by MMD-TBD through reduced clinic visit frequency, lowered transportation costs, and enhanced treatment adherence.

Although our analysis was guided by efforts to identify both converging and diverging perspectives, we found a high degree of alignment between people with TB and HCPs regarding the perceived benefits and implementation considerations (barriers and facilitators) of MMD-TBD. We also reviewed the results across all stakeholder groups to identify areas of convergence and divergence, but did not observe any divergent views. The high degree of stakeholder alignment found in our study indicates that MMD-TBD addresses widely experienced barriers in rural TB service delivery. This convergence may reflect a shared understanding of the practical challenges in TB care and a collective appreciation of the potential value of MMD-TBD. Furthermore, the convergence suggests that implementation challenges may be more operational than conceptual, focusing on developing clear protocols, ensuring adequate training, and establishing robust monitoring systems rather than overcoming fundamental resistance to the approach. This alignment may also indicate that MMD-TBD addresses widely experienced barriers in TB service delivery.

### Strengths and limitations

Our study has several strengths. This study is among the few to propose MMD-TBD for people with TB in rural Uganda and potentially sub-Saharan Africa.

Data collection and analysis were grounded in a well-established theoretical framework, the CFIR. This may result in a theory-informed intervention with high acceptability, appropriateness, and scalability. We gathered data from diverse stakeholders involved in TB care delivery at the regional, district, and health facility levels, ensuring that the findings are both context-relevant and policy-aligned. Additionally, data were collected from people with TB, the direct beneficiaries of MMD-TBD, and their treatment supporters, further enhancing the acceptability and applicability of the findings. Saturation was reached during data collection, ensuring adequate depth for drawing valid conclusions.

Despite the study’s strengths, there are limitations. The generalizability of findings to other contexts may be constrained due to differences in social, economic, policy, and environmental factors. The richness of the data depended on the expertise and openness of the participants, which may have been influenced by social desirability bias. To address these limitations, experienced qualitative research assistants employed rigorous probing techniques, ensuring comprehensive and reliable insights. Concerns about undefined eligibility criteria for MMD-TBD will be addressed by aligning intervention delivery with the elements of differentiated service delivery (DSD) models in Uganda. This approach will tailor MMD-TBD to the clinical characteristics of people with TB—whether stable, unstable, or complex—and to specific populations such as adults, children, and adolescents, as well as contextual factors like rural or urban settings and environmental stability. The uncertain effect of MMD among HCPs compared to routine care will be addressed through a forthcoming noninferiority randomized trial. The trial will align with Uganda’s DSD guidelines for TB and HIV, which emphasize the need for evidence to support differentiated care. As the next step, we will conduct a trial to compare the effectiveness of MMD-TBD with routine care. The 4-visit schedule of MMD-TBD will be assigned to stable people with TB, while the 5-visit schedule will be assigned to those considered unstable, very sick, or diagnosed with bacteriologically confirmed pulmonary TB.

Additionally, HCPs provided more detailed perspectives on MMD-TBD, particularly regarding its timing, frequency, potential benefits, as well as the barriers and facilitators to its implementation. This is understandable, given that MMD is a relatively novel concept and many people with TB have not yet been exposed to it. However, people with TB offered richer insights into their motivation and readiness for MMD-TBD, as well as specific benefits reflected in certain sub-themes within the CFIR domains. This nuance of perspectives should be considered when interpreting the findings.

No barriers were identified within the inner setting or outer setting CFIR domains, likely because participants had limited engagement with organizational or policy-level factors, and the interview guide focused more on individual-level experiences with MMD-TBD. As a result, the absence of data in these domains should not be interpreted as an oversight, but rather as a reflection of the scope and focus of both participant roles and the interview content. Further exploration of these domains will be needed in future research.

## Conclusion and recommendations

The study findings demonstrate that MMD-TBD is perceived to be a feasible and beneficial approach for both HCPs and people with TB. The most preferred MMD option was a 4-visit schedule, with a 5-visit schedule preferred for people with complex health conditions. MMD-TBD not only reduces HCPs’ workload but also offers significant advantages for people with TB, including reduced travel costs, time savings, increased convenience, reduced stigma, and improved treatment adherence. Facilitators included integration with existing treatment models, CHW support (along with family-level treatment support), steady drug availability, and the readiness of HCPs and people with TB for MMD-TBD implementation. Barriers included undefined eligibility criteria, uncertain effects of MMD, and differences in ART and TB drug refill schedules among people with TB/HIV. These barriers should be addressed to maximize the effectiveness of MMD. Further studies to measure the impact of MMD-TBD on treatment outcomes should leverage facilitators and address barriers to adoption and effectiveness.

## Supporting information

S1 File Data Collection Instruments. (PDF)

S2 File Codebook. (DOCX)

S3 File Consolidated Reporting of Qualitative Studies (COREQ) guideline. (PDF)

S1 Checklist Inclusivity in global health research. (DOCX)
