# Supplementary material for: Appropriateness, barriers, and facilitators of multi-month dispensing of tuberculosis drugs in rural eastern Uganda: A qualitative study to inform a non-inferiority randomized trial
Source: PLOS Glob Public Health. 2025 Sep 5;5(9):e0004539. doi: 10.1371/journal.pgph.0004539 (PMC12412997; doi:10.1371/journal.pgph.0004539)
Supplement: S1 File — (PDF) [file pgph.0004539.s001.pdf]

APPENDIX 5: IN-DEPTH INTERVIEW (IDI) GUIDE FOR PEOPLE WITH TB AND TREATMENT SUPPORTERS

**Study title**	**Effectiveness of Multi-Month Dispensing of Anti-Tuberculosis Drugs (MULTI-DAT) Versus Standard of Care on Treatment Success Rate Among People with Drug Susceptible Tuberculosis in Rural Eastern Uganda**
**Short title**	Multi-MOnth Refill of Anti-TB Drugs (MORAD) study
**Sponsor**	Center for Effective Global Action (CEGA) through funding from the National Institutes of Health (NIH).
**Protocol version**	Version #1, January 2024.

Dear Sir/Madam, I thank you for accepting to participate in this interview. Our interview will focus on a new strategy for delivering tuberculosis (TB) treatment. This study seeks to establish whether refilling people with TB for more after the first month of treatment would not produce worse treatment outcomes compared to the existing monthly refills. In the proposed option, people with TB are refilled as usual (bi-weekly) for the first 4 weeks, then monthly for a month, and every two months until treatment is complete. People with TB will visit the healthcare facility 5 times instead of the usual 8 times. Therefore, I will be asking for your reviews regarding it's the relevance and appropriateness of the proposed approach to anti-TB refill. Before we get started, **I would like to show you the proposed strategy in Table 1** below:

Table 1: Timelines for the administration of anti-TB drugs under SOC and MULTI-DAT.

**Type of care**	**Intensive phase**				**Continuation phase**			
Timing of refills	2 weeks	2 weeks	2 weeks	2 weeks	3 mo	4 mo	5 mo	6 mo
SOC	XX	XX	XX	XX	XX	XX	XX	XX
MULTI-DAT	XX	XX	XX		XX		XX	

Note: X detonates duration of anti-TB refill; Green color denotes MULTI-DAT time points.

Participant characteristics and study site

Level of participant	Type of participant
1. Health facility	1. A person with TB.<br>2. Treatment supporter
Duration of interview	Start time of KII __________End time of KII ______________
Age in years: ____________<br><br>Sex<br>1. Male<br>2. Female	Level of education<br>1. None<br>2. Primary<br>3. Secondary<br>4. Tertiary and higher

Topic 1: Appropriateness and relevance of multi-month dispensing of anti-TB drugs

Question 1: Let us talk about how useful and important dispensing TB medications for more than one month would be for people with TB and their treatment supporters. In your opinion how relevant and appropriate is it to dispense TB medications for more than one month?

Probing questions:

- *If appropriate and relevant, why is that the case? How beneficial is it to you?*
- *If not appropriate and relevant, why is it so? What are the likely harms and how likely is it to happen? What aspects of dispensing TB medications for more than one month makes it not goodt? How do you think this aspect can be addressed?*

Topic 2: Characteristics of multi-month dispensing of anti-TB drugs

Question 2: After 4 weeks of TB treatment, this study proposes to dispense TB medications for more than one month on two occasions, making people with TB make 5 health facility visits instead of the usual 8 visits. What aspects of dispensing TB medications for more than one month might make it a successful approach?

Probing on the following:

- *Aspects that make it more attractive to patients and healthcare workers and why.*
- *Whether it will be regarded to have more advantages than the current refill approach.*
- *Aspects of dispensing TB medications for more than one month that need change, and perhaps why?*
- *Would you consider the proposed refill approach simple or complex, and why?*
- *Aspects that do not require change before testing it, including how and why.*
- *Whether the refill approach is considered important and why.*
-
- *Aspects that require change, including how and why.*

Topic 3: Inner setting (Factors within the healthcare system).

Question 3: The dispensing of TB medications for more than one month will be implemented across selected health facilities. Tell me what you think would influence its implementation either positively or negatively within the health facilities.

Probe the following:

- *Factors that might lead to better implementation and how. For example, reduced work load for healthcare workers, positive reception about the refill approach, high motivation among healthcare providers to implement it etc.*
- *Factors that might lead to poor implementation and how. For example, provider attitudes, insufficient health education, high patient numbers leading to delays, reluctance among health workers, etc.*
- *Concerns patients might have with healthcare workers or the health facility.*

Topic 4: Outer setting (Factors outside the healthcare system.

Question 4: With dispensing of TB medications for more than one month, other factors within your community might influence the implementation. Could you share with me what you think these external factors might be?

Probe the following:

- *Family and adherence support measures and how they influence uptake of dispensing of TB medications for more than one month.*
- *How likely social factors such as alcohol consumption might influence adherence to multi-refil.*

Topic 5: Characteristics of people with TB that might influence implementation.

Question 5: Dispensing of TB medications for more than one month is a strategy for people with TB. What would you say will be some of the patient concerns with this approach?

Probe the following:

- *Whether age, sex, residence, and level of education might have an influence.*
- *Whether previous experiences with long-term medication and adherence experience might have an influence.*
- *Whether patients will be more likely or less likely to adhere to medications after refill.*
- *Whether approach works best for people with experience with long-term treatments such as diabetes mellitus, HIV, etc.*

Topic 6: Issues with the implementation process.

Question 6: Let us talk about how dispensing TB medications for more than one month will be implemented. What do you think would make the refill approach work for you?

Probe the following:

- *Whether the time of initiation of the refill is appropriate for patients;*
- *Likely approaches for better implementation at the health facility level;*
- *Likely kinds of patient preparations needed before refil is started.*

Topic 7: Participant's overall assessment.

Question 7: What is your overall impression regarding the relevance of dispensing TB medications for more than one month to patients, healthcare providers, and the healthcare system?

Probe the following:

- *Whether it would be acceptable or not.*
    - *If acceptable, by who and why?*
    - *If not acceptable, how can the intervention be made acceptable?*

APPENDIX 6: KEY INFORMANT INTERVIEW (KII) GUIDE FOR HEALTHCARE WORKERS

**Study title**	**Effectiveness of Multi-MOnth Dispensing of Anti-Tuberculosis Drugs (MULTI-DAT) Versus Standard of Care on Treatment Success Rate Among People with Drug Susceptible Tuberculosis in Rural Eastern Uganda**
**Short title**	Multi-MOnth Refill of Anti-TB Drugs (MORAD) study
**Sponsor**	Center for Effective Global Action (CEGA) through funding from the National Institutes of Health (NIH).
**Protocol version**	Version #1, January 2024.

Dear Sir/Madam, I thank you for accepting to participate in this interview. Our interview will focus on a new strategy for delivering tuberculosis (TB) treatment. This study seeks to establish whether refilling people with TB for more after the first month of treatment would not produce worse treatment outcomes compared to the existing monthly refills. In the proposed option, people with TB are refilled as usual (bi-weekly) for the first 4 weeks, then monthly for a month, and every two months until treatment is complete. People with TB will visit the healthcare facility 5 times instead of the usual 8 times. Therefore, I will be asking for your reviews regarding it's the relevance and appropriateness of the proposed approach to anti-TB refill. Before we get started, **I would like to show you the proposed strategy in Table 1** below:

Table 1: Timelines for the administration of anti-TB drugs under SOC and MULTI-DAT.

Type of care	Intensive phase				Continuation phase			
Timing of refills	2 weeks	2 weeks	2 weeks	2 weeks	3 mo	4 mo	5 mo	6 mo
SOC	XX	XX	XX	XX	XX	XX	XX	XX
MULTI-DAT	XX	XX	XX		XX		XX	

Note: X detonates duration of anti-TB refill; Green color denotes MULTI-DAT time points.

Participant characteristics and study site

Level of participant	Type of participant	
1. National<br>2. Regional<br>3. District<br>4. Health facility	1. NTLP Program. Manager/Commissioner<br>2. NTLP Head of Laboratory Services.<br>3. Director of Clinical Services.<br>4. Regional TB Focal Person.	5. District Health Officer<br>6. District TB and Leprosy Supervisors<br>7. District Laboratory Focal Persons<br>8. TB Focal Persons
Duration of interview	Start time of KII ______________ End time of KII _______________	

Demographic data

Age in years: ___________________<br><br>Years of work experience:<br>________________________________	Sex<br>1. Male<br>2. Female	Highest level of qualification<br>1. Certificate<br>2. Diploma<br>3. Bachelor's degree<br>4. Master's degree<br>5. Ph.D.

Topic 1: Appropriateness and relevance of multi-month dispensing of anti-TB drugs

Question 1: Let us talk about how useful and important multi-month dispensing of anti-TB drugs would be for people with TB and the healthcare system. What is your opinion regarding the appropriateness and impact of the proposed approach to delivering TB medications to patients?

Probing questions:

- *If appropriate and relevant, why is that the case? How beneficial is it?*
- *If not appropriate and relevant, why is it so? What are the harms and how likely is it? what aspects intervene not fit? How do we address this aspect?*

Topic 2: Characteristics of multi-month dispensing of anti-TB drugs

Question 2: Following 4 weeks of anti-TB refill, the study proposes to refill TB drugs for 1 month and then 2 months on two occasions. This would make the patient visit the health facility 5 times instead of 8. What would you say are the aspects of this approach that will make it more successful?

Probing on the following:

- *Whether approach might be regarded simple or complex, and in what ways.*
- *Whether it might be appealing to patients and healthcare workers and why.*
- *Whether the timing and frequency of refills are adequate.*
- *Aspects that require change and why? Aspects that do not require change.*

Topic 3: Inner setting (Factors within the healthcare system).

Question 3: The intervention will be implemented across selected health facilities. Tell me what you think would influence the implementation of the intervention whether positively or negatively

Probe the following:

- *Factors that might lead to better implementation of the refil approach such as leadership support at the health facility, healthcare provide attitudes and perceptions, prioritization of the approach, patient education, etc.*
- *Factors that might lead to poor implementation, for example no buy-in from health workers.*
- *Concerns among healthcare workers about the advantages in improving treatment outcomes.*

<u>Topic 4: Outer setting (Factors outside the healthcare system.</u>

<u>Question 4:</u> Even if multi-month dispensing of anti-TB drugs is implemented at the health facility level, there might be external factors which might influence the implementation. What are your views regarding external factors that might influence the implementation of multi-month dispensing of anti-TB drugs

Probe the following:

- *Treatment support systems at family and community levels.*
- *Patient behaviours such as alcohol consumption, treatment adherence, etc.*
- *The likeliness of the factors affecting the success of the approach.*

<u>Topic 5: Characteristics of people with TB that might influence implementation.</u>

<u>Question 5</u>: Multi-month dispensing of anti-TB drugs is a strategy for people with TB. What do you think are some of the patient factors that might influence the implementation of multi-month dispensing of anti-TB drugs?

Probe the following:

- *Possibility of patient factors such as age, sex, residence, and level of education influencing the success of the refil approach.*
- *Likelihood of patients adhering to the treatment.*
- *Whether the refill approach would better fit best for people on longer term medication use such as people living with HIV compared to people without HIV, diabetic versus non-diabetic, etc.*

<u>Topic 6: Issues with the implementation process.</u>

<u>Question 6:</u> Let us talk about the implementation of multi-month dispensing of anti-TB drugs. What do you think would make the implementation work?

Probe the following:

- *Whether the time of initiation of the refill is appropriate for patients.;*
- *Strategies for better implementation, monitoring, and evaluation at the health facility level;*
- *Patient and healthcare provider preparations, motivations, and monitoring..*

<u>Topic 7: Participant's overall assessment.</u>

Question 7: What is your overall impression regarding the relevance of the multi-month dispensing of anti-TB drugs for patients, healthcare providers, and the healthcare system?

Probe the following:

- *Whether multi-month refill is acceptable or not.*
    - *If acceptable, why?*
    - *And, if not acceptable, why and how it can be made cceptable?*
