# Supplementary material for: Appropriateness, barriers, and facilitators of multi-month dispensing of tuberculosis drugs in rural eastern Uganda: A qualitative study to inform a non-inferiority randomized trial
Source: PLOS Glob Public Health. 2025 Sep 5;5(9):e0004539. doi: 10.1371/journal.pgph.0004539 (PMC12412997; doi:10.1371/journal.pgph.0004539)
Supplement: S2 File — (DOCX) [file pgph.0004539.s002.docx]

**S2 File: Codebook**

| **CFIR constructs (Themes)** | **Facilitator sub-themes—*factors that participants identified as supporting the implementation of multi-month dispensing of TB drugs.*** | **Illustrative quotes** |
| --- | --- | --- |
| **Intervention Characteristics** | Integration with existing treatment models | *If you look at the schedule, which is here, at least you see a patient 5 times in 6 months, which I think is good. And, also, I think it aligns well with the multi-month schedule for HIV, which we are doing now.*  *The good thing is that it [MMD of TB drugs] is coming from a background that is already appreciated, so is not going to be difficult to implement this one. The backbone is already laid, as people know that treatment is 6 months. So, it is not going to be difficult.* |
|  | Person-centeredness of MMD | *To the clients [people with TB], this approach [MMD of TB drugs] is patient-centered care so the issue of transport costs is reduced. And, the other bit also, is that we give clients a responsibility to take care of themselves away from us [the HCPs] a little.* |
|  | Practical supporting evidence | *It (MMD of TB drugs) will be effective. Because, if I can remember during the time of the COVID-19 pandemic, we were refilling the clients for 1 month, even 2 months, even 3 months, and they were very okay. It improved their appointment-keeping and treatment adherence and even reduced their transport costs.*  *Even I have tried [MMD of TB drugs] personally, although it was not in the treatment guideline or it was not stated in any way. But we have been trying and you find the books would get rejected. You would find the dispenser trying to reduce the period.* |
| **Outer Setting** | Presence of CHWs for decentralized support | *Another option for follow-up can be the use of the VHTs [Village Health Teams]. So, the nearby VHT can follow up on this patient and then, complies the reports and send them to the health center every month. So monthly VHT reports should include follow-up information about patients on TB treatment.*  *We need to strengthen CB-DOTS [community-based Directly Observed Therapy Short Course] in the community by using relatives. For example, if a woman is sick, a man can be a [treatment] supporter. They call them treatment supporters.* |
|  | Family-level treatment support and engagement | *The only factor I know is how the patients [people with TB] take drugs at home. If we get treatment supporters who are good and willing to do their work and observe them swallow their drugs, there will be no big challenge.*  *We need to commit the treatment observers [treatment supporters] and ensure they understand, and the patients must also understand because this is a protracted treatment. So, somebody could easily relax.*  *For me who is giving the drugs to my child [as a mother], I will ensure that the child takes the drugs on time. If I am not at home, I will call someone at home to ensure that the child is given the drugs on time because it will not be good for his health if he does not swallow [the TB drugs].* |
| **Inner setting** | Steady availability of drugs | *The most important thing is having the drugs available. So, for it [MMD of TB drugs] to be successful, then facilities should be stocked with enough drugs because the challenge sometimes that we face is drug stock out. So, you cannot plan to give a refill of 2 months when you have little stock. So, we must have enough stock available for us to be able to, successfully implement it.*  *Now that if we adopted this method, then the stocks have to be sufficient on the ground. At the end of the day, if we give one client TB drugs for 2 months, yet even when we have been given 2 weeks, 1 month, we have been having challenges. But recently, I am beginning to realize that the drug stockouts are steady. I am only praying that as we take up this kind of approach [MMD of TB drugs], we should also be working seriously on the supply to be on time and sufficient.* |
|  | Operational guidelines (MMD protocols and procedures) | *The way I know, it will have to be like a policy. The Minister of Health [MoH] has to produce a standard operating procedure (SOP) whereby health workers are supposed to follow. But if SOPs are not there, we cannot achieve what we want. Because in everything in health, we have SOPs and if SOPs are not there, we shall not achieve.* |
|  | Medication instructions | *Maybe they [HCPs] can give us some write-up [medication instructions] so that we can also be reading on what you're supposed to be doing. Because sometimes you come and you get a lot of information. And now as you are not medics [HCPs], sometimes you don't get everything or you just get a few things.* |
|  | Enhanced monitoring and evaluation (e.g., revised data collection tools, regular reviews) | *For health workers, even the registers have to be changed because the registers were printed for two weeks' refills. But still, we know the registers are not like that. We were able to show [during MMD] in the register that the person was refilled for one month.*  *I think for those selected health facilities, the tools [data collection tools] equally need to be provided. But if we leave the tools in the way they are, we might not get the real results. So, let those columns in the TB Unit register for client visits be changed to fit MMD.*  *There should be monthly monitoring of data to see who has not come and who has not come. Then to those who have not come, something should be done. At the monthly data review in the health center, these data should be shared or presented to all staff to know that there are patients on treatment and that new changes [MMD of TB drugs] have happened.* |
|  | Clinic accessibility for managing medication side effects and complaints | *I think the health facility should constantly be open to us [accessible health facility] so that if we [people with TB] take these drugs and I develop a problem [side effects], I should be able to come back to the health facility at any time for the health workers to see me.* |
| **Characteristics of individuals** | HCP training | *Before this program takes place [wide implementation of MMD of TB drugs], it has been a long time since when they last trained health workers on TB. We need to organize either at the district level or at the health facility level, serious mentorships or training of the focal persons, plus even the clinical team on TB management.*  *As long as the health worker has knowledge or knows the benefits and the outcomes of the implementation of this program [MMD of TB drugs], it will help him or her to manage patients very well.* |
|  | HCP readiness to implement MMD | *We have no problem with this schedule [of MMD of TB drugs] because it is very good on the patient side and very good on the health worker side. At the health facility, the TB focal person is already there who is supposed to track these patients. Generate a list of patients on TB treatment at different intervals so that that list is tracked up to completion.*  *The health workers will be very interested in ensuring that these patients are given multi-month refills. We are also interested in making sure we monitor the treatment.*  *This is [MMD of TB drugs] even long overdue. We welcome it, and the clients will appreciate it because it will lead to good adherence among our clients [people with TB].* |
|  | Patient motivation and readiness to utilize MMD | *Even as patients [people with TB], we have been thinking about it [MMD of TB drugs] but we have nowhere to take the idea. It has been a tough thing for us to frequently travel to the health facility to collect drugs [TB drugs]. The home is very far away and you know I have to ride a bicycle.*  *I will be happy with the initiative [MMD of TB drugs] and I will even propose that they increase it to 3 months so that in a short time I will finish collecting the drugs [TB drugs].* |
| **Process of Implementation** | Patient engagement and support (e.g., active follow-up, patient reminders, health education, and counseling) | *We [HCPs] can also use the phones to contact the patient directly or the caregiver to see if the patient is cooperating [adhering to medications].*  *We need to strengthen patient education at the facility level. Let the patient understand that at the end of 1 month, he/she is supposed to come for a refill. We need proper counseling of patients [people with TB] so that they can follow up on the treatment very well. Since we are not telling them to come often to the unit [health facility or TB clinics], they need to understand that their lives are at stake if they do not take the medicines.*  *We [HCPs] need to do proper documentation and even put up a schedule since these patients are few. They can even display that for Patient “A”, the return date is this and for Patient “B”, the return date is that. By doing so, we make this program [MMD of TB drugs] succeed.*  *We need to draw up a schedule of visiting clients [people with TB] at regular times based on the refills.* |
|  | Leadership support for MMD implementation | *This [MMD of TB drugs] now needs the NTLP [National TB and Leprosy Control Program] and the Ministry of Health to at least bring up this program [MMD of TB drugs] so that this program can be implemented. Because the program [MMD of TB drugs] is going to work for us very well.*  *We need to bring the leaders on board. And, when I look at the letters [support letters from NTLP support letter and the districts] that you already have here and the people being interviewed, they are people at the heart of TB management. So, the research is already giving us a direction. I feel that the same approach should be used up to the lower level so that the health facility heads [In-charges] are brought on board.* |
| **CFIR constructs** | **Barrier sub-themes—factors that participants identified as hindering the implementation of multi-month dispensing of TB drugs.** | ***Illustrative quotes*** |
| **Intervention characteristics** | Undefined eligibility criteria for MMD | *All in all, giving a multi-month refill is not a bad idea, but then to which category of people are we giving a multi-month refill? I would wish that we first put a specific category of patients on monthly refills and not generalize on all. For those few or those who understand their disease, the approach would be good. As you are interacting with your patient for the first time, you can have a picture of someone who cares for themselves. For those, you can give multi-month refills if they have genuine telephone contact. Those could be the clients [people with TB] for multi-month refills.*  *We should start multi-month for PCDs [persons with pulmonary clinically diagnosed TB] and we keep the PBCs [persons with bacteriologically confirmed pulmonary TB] at the health facility because they are very infectious, and we want to encourage them to ensure that they take their treatment correctly and also get enough information to make them understand that they need to add care on their treatment and take it for their full duration.* |
|  | Skepticism about MMD among HCPs | *We need to look at the strength of the initial schedule where our patients will come every 2 weeks and look at the strength of this new proposed schedule, and then we compare the two approaches. The other one [routine care], the advantage it would give is that there would be timely follow-up physically although it could be so expensive on the patient's side.*  *You need to evaluate it. Is it doable? Is it helping? You need to make a comparison between the new approach and the present one [MMD of TB drugs]. You need to follow it up to see if it is working [asses effectiveness]. Of course, whether it is affecting the TB outcome and what the treatment outcomes are. So, it is just a matter of getting the data comparing the previous [routine care] and the current [MMD of TB drugs] to see if it is beneficial.*  *For every new intervention, it has to be started on a smaller scale. I think we have to wait and see which facilities are beginning, and then we will need to share their challenges and then achievements.* |
|  | Differing refill schedules for TB/HIV co-infection | *The challenge I see there is that HIV refills involve 1 month, 3 months, 6 months, and so on. But when it comes to TB, especially for those who are TB/HIV co-infected, the appointment days will differ. If there's a way of harmonizing the HIV and TB co-infected appointments in line with the TB refills, it will reduce transport costs to our client.*  *What is again important is we have to harmonize TB and HIV refills so that the pill burden and the appointment date match. Because, you know, most ARVs, they are packed for 3 months and one month. And, most of the health facilities I have visited have ARVs packed for 3 months. We can divide the pills, but again, dividing it and how to keep the balance is a challenge.* |
| **Characteristics of individuals** | Non-adherence due to perceived treatment burden and patient decision-making, alcohol consumption, forgetfulness, medication sharing, and health neglect | *Outside the health facility, the patient may mean to relax when gets too many drugs at ago. The patient would say can't this be enough really to cure this [TB disease]? The patient now starts negotiating within himself or herself.*  *One of the worst things that might affect [MMD of TB drugs] will be alcohol drinking because somebody will even forget to take drugs.*  *There's a tendency to forget to take the drugs once you have all of them [TB drugs]. You will forget to swallow them.*  *We have also realized that when some patients are given more drugs, they start to share them with other people or use them for other things because the duration of refill is not calling for their immediate return to the clinic.*  *Someone may not take the drugs once given. This is because some people are careless and do not love themselves. But when we have someone at home to keep advising and encouraging the person, the person will take it.* |
| **Process of Implementation** | Patient disengagement driven by insufficient follow-ups | *This [MMD] needs some careful implementation and follow-up so that it does not lead us to high loss to follow-up and [treatment] failure rates because, without the proper follow-up of these patients, they may come back when they are worse than the way we started [on treatment]* |
